# Cyclin D1 overexpression and poor clinical outcomes in Taiwanese oral cavity squamous cell carcinoma

**DOI:** 10.1186/1477-7819-10-40

**Published:** 2012-02-16

**Authors:** Shiang-Fu Huang, Sou-De Cheng, Wen-Yu Chuang, I-How Chen, Chun-Ta Liao, Hung-Ming Wang, Ling-Ling Hsieh

**Affiliations:** 1 Department of Otolaryngology, Head and Neck Surgery, Chang Gung Memorial Hospital, Tao-Yuan, Taiwan; 2 Department of Anatomy, Chang Gung University, Tao-Yuan, Taiwan; 3 Department of Pathology, Chang Gung Memorial Hospital, Tao-Yuan, Taiwan; 4 Division of Hematology/Oncology, Department of Internal Medicine, Chang Gung Memorial Hospital, Tao-Yuan, Taiwan; 5 Department of Public Health, Chang Gung University, Tao-Yuan, Taiwan

**Keywords:** oral squamous cell carcinoma, lymph node metastasis, cyclin D1

## Abstract

**Background:**

Cyclin D1 gene regulates cell cycle and plays an important role in the tumorigenesis of human cancers. The association between cyclin D1, clinicopathologic parameters and prognosis in oral cavity squamous cell carcinoma (OSCC) is inconclusive.

**Methods:**

A total of 264 male OSCCs were examined for cyclin D1 protein expression using immunohistochemistry (IHC). The expression levels of cyclin D1 were defined as overexpression when more than 10% of tumor cells displayed nuclear staining with moderate to strong intensity.

**Results:**

Overexpression of cyclin D1 was found in 97 (36.7%) OSCCs. Cyclin D1 protein overexpression was significantly associated with lymph node metastasis (P = 0.002), tumor cell differentiation (P = 0.031) and tumor stage (P = 0.051), but not associated with age onset, cigarette smoking, alcohol drinking, or areca quid chewing. Overexpression of cyclin D1 was also significantly associated with poor clinical outcomes in terms of disease-free survival (DFS, P = 0.002) and overall survival (OS, P < 0.001). The effects of cyclin D1 protein overexpression on DFS (hazard ratio (HR) = 1.540; 95% confidence interval (CI), 1.068 - 2.222) and OS (HR = 1.702; 95% CI, 1.168 - 2.480) were still existed after adjusting for clinicopathological paremeters (such as age, primary tumor status, tumor cell differentiation, and lymph node metastasis) using logistic multivariate analysis.

**Conclusion:**

Cyclin D1 protein worked as an independent prognostic factor and can be as a biomarker for the aggressiveness of OSCC.

## Background

In Taiwan, oral cancer is the fourth most common cancer in men [1]. Epidemiologic studies have shown that environment and personal habits, particularly tobacco use, areca quid (AQ) chewing and alcohol abuse, are major etiologic factors in the induction and progression of this disease. About two thirds of oral cancers were occurred in oral cavity. The primary treatment for oral cavity squamous cell carcinoma (OSCC) is radical surgery with or without post-operative chemoradiation [2]. However, for patients with tumors at advanced stage, their prognoses are usually discouraging. If we can better understand the characteristics of OSCCs, this may ultimately help clinicians to provide OSCC patients with more appropriate treatment.

The cyclin D1 gene (*CCND1*) located on chromosome 11q13 is a positive regulator of the cell cycle. It encodes a nuclear protein that forms complexes with cyclin-dependent kinases 4 and 6, which phosphorylate and inactivate the retinoblastoma protein (pRb). Inactivation of pRb allows cell cycle progression from G1 to S phase [3]. Although it has been shown that increased expression of cyclin D1 caused potential for growth advantage and enhances tumorigenesis [4,5], the role of cyclin D1 as a prognostic marker in OSCC remains controversial. Overexpression of cyclin D1, was reported to be associated with recurrence and shortened overall survival in operable cases of squamous cell carcinoma of head and neck (SCCHN) [6-8]. However, a study of 45 patients with a significantly higher proportion of oral carcinoma found no significant correlation between overexpression of cyclin D1 and any of the clinicopathological parameters studied [9]. Therefore, this study was designed to investigate the correlation of cyclin D1 expression with clinicopathologic parameters and disease outcome in 264 Taiwanese male OSCC patients.

## Materials and methods

### Patients and clinical diagnosis

This study was approved by the Institutional Review Board of Chang Gung Memorial Hospital. Two hundred and sixty-four male Taiwanese oral cancer patients received radical surgery prior to any treatments during March 1999 and December 2005 at Chang Gung Memorial Hospital, Lin-Kuo, were recruited for participation in this study. All patients gave informed consent for participation and were interviewed uniformly before surgery by a well-trained interviewer. The questionnaire used in the interview sought detailed information on general demography as well as current and past cigarette smoking, alcohol drinking and AQ chewing habits. If the patients had ever smoked cigarette, chewed AQ and drank alcohol on a regular basis (at least once a week for 1 year) were classified as tobacco, AQ and alcohol users, respectively. For each patient, clinical histological parameters (ie, pT classification including skin and bone invasion, differentiation, nodal-status, lymph node extracapsular spread (ECS), and perineural invasion) were reviewed by the pathologist and scored according to the recommendations for the reporting of specimens containing oral cavity and oropharynx neoplasms by the Association of Directors of Anatomic and Surgical Pathology [10].

### Immunohistochemical analysis

**Figure 1:**
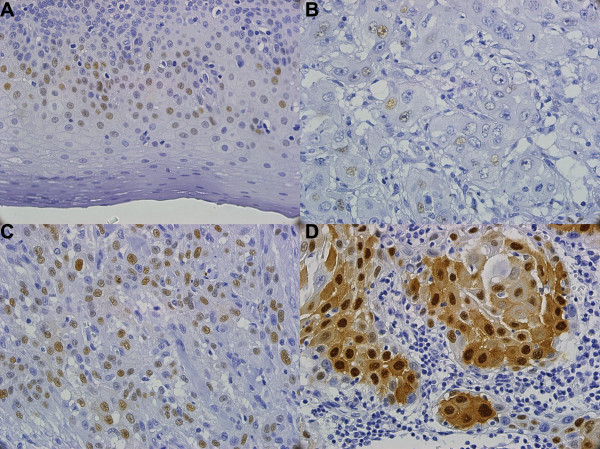
**Immunohistochemistry staining patterns of cyclin D1 in tissues from oral squamous cell carcinoma (400×)**. A. Tumor-normal adjacent with weak intensity, B. Tumor with weak intensity, C. Tumor with moderate intensity, and D. Tumor with strong intensity.

Immunohistochemical staining for cyclin D1 protein was performed as described previously [11]. Briefly, the paraffin embedded tumor sections (5 μm) were deparaffinized, retrieved with heat in 10 mM citrate buffer (pH 6.0) and treated with 3% hydrogen peroxide to remove endogenous peroxidase activity. Anti-cyclin D1 monoclonal antibody SP4 (1:200) (Lab Vision, Fremont, CA) was used as the primary antibody and the NovoLink™ Polymer Detection System (Novocastra Laboratories Ltd., Newcastle Upon Tyne, UK) was used as the detection system. The slides were then counterstained with hematoxylin and coverslipped with Permount and examined for the extent and intensity of nuclear and non-nuclear staining in tumor cells and for background staining by the pathologist (WYC) in a blind manner. In the present study, when more than 10% of cells displayed nuclear staining [7,12,13] and intensity scores of moderate and strong, then there was considered to be cyclin D1 overexpression (Figure 1).

### Statistical analysis

Statistical analysis was performed using the SPSS statistical package (SPSS, Chicago, IL). The correlations between the cyclin D1 status and age, TNM stage, cigarette smoking, alcohol drinking, and AQ chewing was examined by χ^2 ^test or Fisher's exact test. Survival curves were constructed by the Kaplan-Meier method and the curves were compared using the log-rank test. The Cox regression model was applied to adjust simultaneously all potential prognostic variables including age, primary tumor status, tumor cell differentiation, and lymph node metastasis. A two-sided value of *p *< 0.05 was considered statistically significant.

## Results

### Patient characteristics

The clinicopathological features of the 264 OSCC male patients who took part in this study are listed in Table 1. The major primary sites were the bucca (40.2%, 106/264) and the tongue (37.9%, 100/264). Overall, 85.2% (225/264) of the patients were cigarette smokers, 50.4% (133/264) were alcohol drinkers and 86.0% (227/264) were AQ chewers. The primary treatment for these 264 patients was surgery and 124 (47.0%) and 59 (22.3%) of the patients undergoing additional radiation therapy and chemoradiotherapy, respectively. The median follow-up was 46.5 months.

**Table 1:** Characteristics of the 264 OSCC patients

Characteristic	
*Age (year)*	
Mean ± SD	49.33 ± 11.01
Range	26-78
*Site of primary tumor [No. of patients (%)]*	
Tongue	100 (37.9)
Bucca	106 (40.2)
Others*	58 (22.0)
*Pathologic stage [No. of patients (%)]*	
Stage I	18 (6.8)
Stage II	56 (21.2)
Stage III	53 (20.1)
Stage IV	137 (51.9)
*AQ chewing [No. of patients (%)]*	
Yes	227 (86.0)
No	37 (14.0)
*Cigarette smoking [No. of patients (%)]*	
Yes	225 (85.2)
No	39 (14.8)
*Alcohol drinking [No. of patients (%)]*	
Yes	133 (50.4)
No	131 (49.6)

SD: standard deviation; AQ: areca quid. *Including mouth floor (n = 10), lip (n = 6), alveolar ridge (n = 26), hard palate (n = 5) and retromolar trigone (n = 11).

### Prognostic implications of cyclin D1 protein expression

Overexpression of cyclin D1 was found in 97 (36.7%) OSCCs. As shown in Table 2, cyclin D1 overexpression was more prevalent in tumors at advanced stage than those at early stage (*p *= 0.051) and moderate/poor differentiation than well differentiation (*p *= 0.031). More specifically, tumors with characteristics of lymph node metastasis and lymph node ECS had significantly higher frequency of cyclin D1 overexpression than tumors without those characteristics (*p *= 0.002). The frequency of cyclin D1 overexpression was lower in buccal cancer than other subsites (*p *= 0.007). On the other hand, cyclin D1 protein overexpression was not associated with age, primary tumor status, skin invasion, bone invasion, perineural invasion, cigarette smoking, AQ chewing, and alcohol drinking.

**Table 2:** The associations between cyclin D1 overexpression and clinicopathological parameters (N = 264)

	Cyclin D1 protein overexpression		
	No [N (%)]	Yes [N (%)]	*p *value
Age			
< 50 yrs (n = 151)	93 (61.6)	58 (38.4)	0.516
≥ 50 yrs (n = 113)	74 (65.5)	39 (34.5)	
Subsites			
Tongue (n = 100)	52 (52.0)	48 (48.0)	**0.007**
Bucca (n = 106)	81 (76.4)	25 (23.6)	
Others (n = 58)	34 (58.6)	24 (41.4)	
Tumor stage			
Early (n = 76)	55 (72.4)	21 (27.6)	**0.051**
Advanced (n = 188)	112 (59.6)	76 (40.4)	
Primary tumor			
T1/T2 (n = 129)	77 (59.7)	52 (40.3)	0.240
T3/T4 (n = 135)	90 (66.7)	45 (33.3)	
Differentiation			
Well (n = 107)	76 (71.0)	31 (29.0)	**0.031**
Moderate/poor (n = 157)	91 (58.0)	66 (42.0)	
Tumor depth			
< 10 mm (n = 95)	66 (69.5)	29 (30.5)	0.116
≥ 10 mm (n = 169)	101 (59.8)	68 (40.2)	
Lymph node metastasis			
LN (-); ECS (-) (n = 138)	101 (73.2)	37 (26.8)	**0.002**
LN (+); ECS (-) (n = 55)	30 (54.5)	25 (45.5)	0.001*
LN (+); ECS (+) (n = 71)	36 (50.7)	35 (49.3)	
Skin invasion			
Yes (n = 28)	22 (78.6)	6 (21.4)	0.075
No (n = 236)	145 (61.4)	91 (38.6)	
Bone invasion			
Yes (n = 67)	42 (62.7)	25 (37.3)	0.911
No (n = 197)	125 (63.5)	72 (36.5)	
Perineural invasion			
Yes (n = 72)	39 (54.2)	33 (45.8)	0.061
No (n = 192)	128 (66.7)	64 (33.3)	
AQ chewing			
Yes (n = 227)	147 (64.8)	80 (35.2)	0.210
No (n = 37)	20 (54.1)	17 (45.9)	
Cigarette smoking			
Yes (n = 225)	147 (65.3)	78 (34.7)	0.093
No (n = 39)	20 (51.3)	19 (48.7)	
Alcohol drinking			
Yes (n = 133)	83 (62.4)	50 (37.6)	0.772
No (n = 131)	84 (64.1)	47 (35.9)	

LN: lymph node metastasis; ECS: extracapsular spread; AQ: areca quid. * χ2 trend test.

**Figure 2:**
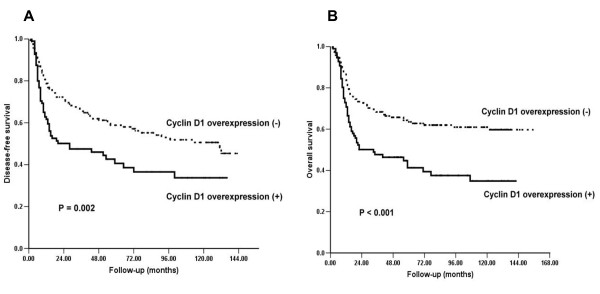
**Kaplan-Meier curves for disease-free survival (DFS) and overall survival (OS) based on analysis of cyclin D1 protein overexpression**. (A) Cyclin D1 overexpression was significantly associated with disease-free survival. (B) Cyclin D1 overexpression was significantly associated with overall survival.

Disease-free and overall survival (DFS and OS) were significantly worse in patients that were cyclin D1 overexpressed in the tumors when compared with patients that were cyclin D1 not overexpressed (Figure 2). To assess the effect of other clinicopathological factors on DFS and OS, a Cox model was carried out initially using univariate analysis. As shown in Table 3, primary tumor status (*p *= 0.010), differentiation (*p *= 0.020), and lymph node metastasis (*p *< 0.001) were significantly associated with a poorer OS. Lymph node ECS (*p *< 0.001) was also significantly associated with a poorer DFS. After adjusting for age, tumor cell differentiation, primary tumor status and lymph node metastasis using multivariate analysis, cyclin D1 overexpression was still associated with a significantly increased hazard ratio for DFS and OS, respectively, of 1.540 (95% CI, 1.068 - 2.222) and 1.702 (95% CI, 1.168 - 2.480) compared with the patients with tumors without cyclin D1 overexpression (Table 4).

**Table 3:** Univariate regression model of prognostic covariates in 264 patients with oral cavity squamous cell carcinoma regarding disease-free and overall survival

	DFS		OS	
Characteristic	HR (95% CI)	P value	HR (95% CI)	P value
Age (years old)				
< 50	1		1	
≥ 50	1.038 (0.731 - 1.472)	0.836	1.118 (0.779 - 1.605)	0.545
Differentiation				
Well	1		1	
Moderate/poor	1.232 (0.864 - 1.758)	0.250	1.571 (1.074 - 2.300)	**0.020**
Primary tumor				
T1/T2	1		1	
T3/T4	1.301 (0.918 - 1.843)	0.138	1.617 (1.123 - 2.328)	**0.010**
Nodal status				
(-) metastasis, (-) ECS	1		1	
(+) metastasis, (-) ECS	1.042 (0.639 - 1.699)	0.870	1.349 (0.813 - 2.238)	0.247
(+) metastasis, (+) ECS	2.550 (1.726 - 3.765)	**< 0.001**	3.391 (2.265 - 5.077)	**< 0.001**
Overexpression of cyclin D1 protein				
No	1		1	
Yes	1.710 (1.202 - 2.434)	**0.002**	1.970 (1.371 - 2.831)	**< 0.001**

DFS: disease-free survival; OS: overall survival; HR: hazard ratio; CI: confidence interval; ECS: extracapsular spread.

**Table 4:** Multivariate Cox regression model of prognostic covariates in 264 patients with oral cavity squamous cell carcinoma regarding disease-free and overall survival

	DFS		OS	
Characteristic	HR (95% CI)	P value	HR (95% CI)	P value
Age (years old)				
< 50	1		1	
≥ 50	1.076 (0.755 - 1.534)	0.685	1.108 (0.769 - 1.595)	0.582
Differentiation				
Well	1		1	
Moderate/poor	1.092 (0.759 - 1.571)	0.636	1.334 (0.904 - 1.968)	0.146
Primary tumor				
T1/T2	1		1	
T3/T4	1.176 (0.822 - 1.684)	0.375	1.462 (1.005 - 2.127)	**0.047**
Nodal status				
(-) metastasis, (-) ECS	1		1	
(+) metastasis, (-) ECS	0.929 (0.562 - 1.536)	0.775	1.128 (0.672 - 1.892)	0.649
(+) metastasis, (+) ECS	2.199 (1.446 - 3.343)	**< 0.001**	2.627 (1.711 - 4.035)	**< 0.001**
Overexpression of Cyclin D1 protein				
No	1		1	
Yes	1.540 (1.068 - 2.222)	**0.021**	1.702 (1.168 - 2.480)	**0.006**

DFS: disease-free survival; OS: overall survival; HR: hazard ratio; CI: confidence interval; ECS: extracapsular spread.

## Discussion

The clinical outcome of OSCC patients is strongly influenced by the stage of disease, particularly with positive nodal metastasis or lymph node ECS [14]. In the present study, we found that lymph node metastasis with positive lymph node ECS was the major determinant of OSCC outcomes (Table 3 and 4) and overexpression of cyclin D1 was significantly associated with lymph node metastasis (Table 2). In addition, OSCC survival was significantly influenced by the level of cyclin D1 expression (Figure 2a and 2b) and the relationship still exists in the multivariate analysis after adjusting for age and lymph node metastasis (Table 4). Cyclin D1 overexpression frequencies were reported ranging from 17.1% to 83.0% in OSCC [7,8,12,13,15-19]. In the present study, 36.7% of OSCCs showed overexpression of cyclin D1 protein. The reason for such variation is uncertain, but may reflect mainly differences in the primary antibody used and scoring systems applied. In the present study, a rabbit monoclonal antibody SP4 raised against a synthetic peptide from C-terminus of human cyclin D1 was used, which is deemed to be specific to cyclin D1 [20]. Gown et al. [21] have reported that a normalized scoring method that subtracts the score for non-neoplastic cells from that for tumor cells reduces the false-positive rate from 31% to 5% in assessing HER2 IHC results for breast cancer. We found that most normal basal epithelial cells demonstrated cyclin D1 equivocal weak nuclear staining or background staining in the present series. Therefore, we defined that samples with more than 10% of the cells with moderate and strong cyclin D1 intensity were as having a state of overexpression.

Cyclin D1 is one of the key proteins involved in cell cycle control and is essential for G1 to S transition. Cyclin D1 interacts with cyclin-dependent kinase 4/6 and forms a complex that inactivates pRB through phosphorylation, allowing passage through the restriction point and progression through the G1 phase [22]. Dysregulation of cyclin D1 expression or function contributes to the loss of normal cell cycle control during tumorigenesis. Our results indicated the cyclin D1 overexpression was an early event in the tumorigenesis of OSCC.

Previous studies have found a correlation between cyclin D1 overexpression and the presence of regional lymph node metastases in several human tumors, including head and neck SCC [7,19,23]. Our results indicated that the odds ratio of nodal metastases in tumors that overexpressed cyclin D1 was 2.482 (95% CI, 1.484 - 4.149) times of those without overexpression. This suggests that cyclin D1 overexpression may be related to local invasiveness and a more aggressive clinical behavior of OSCC. Lymph node metastasis could thus be the pathway that patients with cyclin D1 overexpression had poor prognosis. To clarify the mechanism of lymph node spread of tumor cells via cyclin D1 overexpression, further study is necessary to explore cyclin D1-dependent molecules which would play an important role in the tumor invasion and metastasis.

In our series, moderately and poorly differentiated tumors demonstrated a significantly higher prevalence of cyclin D1 overexpression than well-differentiated tumors (*p *= 0.031). The relationship was consistent with previous reports [7,13,18]. However, the reason for this relationship remains unclear. Both *in vitro *and *in vivo *studies have found that cyclin D1 overexpression can inhibit the differentiation of myoblasts [9,24] and intestinal epithelial cells [25], thus raising the possibility that cyclin D1 overexpression may play a role in the inhibition of tumor cell differentiation in some cell types [7].

Overexpression of cyclin D1 was also associated with reduced DFS and OS. This is in agreement with several recent publications investigating the prognostic significance of cyclin D1 in OSCC [7,8,13,16,17,19]. In our study, after adjusting the tumor differentiation, primary tumor status and lymph node metastasis, cyclin D1 overexpression still adversely influences the patients' survival. Thus, our data provide further evidence that overexpression of cyclin D1 imparts independently a poor prognosis in OSCC.

## Conclusions

Overexpression of cyclin D1 protein was significantly associated with lymph node metastasis, tumor cell differentiation and tumor stage in Taiwanese OSCC. In addition, cyclin D1 overexpression also worked as an independent prognostic factor, since cyclin D1 overexpression was still significantly associated with poor prognosis both in terms of DFS and OS in the multivariate analysis after adjusting age, primary tumor status, lymph node metastasis and tumor cell differentiation.

## Abbreviations

(OSCC): oral cavity squamous cell carcinoma; (IHC): immunohistochemistry; (DFS): disease-free survival; (OS): overall survival; (HR): hazard ratio; (AQ): areca quid; (pRb): retinoblastoma protein; (SCCHN): squamous cell carcinoma of head and neck; (ECS): extracapsular spread.

## Competing interests

The authors declare that they have no competing interests.

## Authors' contributions

SFH, SDC and LLH conceived the study. WYC performed the scoring of immunohistochemistry staining. SFH, IHC, CTL and HMW collected the cases and clinical information. SFH and LLH interpreted the staining results and performed the statistical analysis. SFH performed the literature review and wrote the manuscript. SDC and LLH supervised the experiments. LLH supervised the manuscript writing. All authors read and approved the final manuscript.

## Acknowledgements

We thank Kai Cheng for assistance with immunohistochemistry staining. This study was supported by Grant CMRPG340381, CMRPG360701, CMRPG360851, CMRPG371511, CMRPG391411, CMRPD190031 and CMRPG3A0281 from Chang Gung Memorial Hospital, Grants NSC94-2314-B-182-030, NSC98-2314-B-182-046-MY3 and NSC99-2314-B-182A-036-MY3 from the National Science Council, Grant DOH99-TD-C-111-006 from Department of Health, Executive Yuan, Taiwan.
